# The ERH gene regulates migration and invasion in 5637 and T24 bladder cancer cells

**DOI:** 10.1186/s12885-019-5423-9

**Published:** 2019-03-12

**Authors:** Kun Pang, Zhiguo Zhang, Lin Hao, Zhenduo Shi, Bo Chen, Guanghui Zang, Yang Dong, Rui Li, Ying Liu, Jie Wang, Jianjun Zhang, Longjun Cai, Xiaoxiao Han, Conghui Han

**Affiliations:** 1 Department of Urology, Xuzhou Central Hospital, Jiangsu Xuzhou Jiefang South Road, No.199, Jiangsu, China; 2 Department of Urology, The third affiliated hospital of Soochow University, No.185, Juqian Street, Changzhou City, Jiangsu Province China; 3 College of Life Sciences, Jiangsu Normal University, 101 Shanghai Road, Tongshan New District, Xuzhou City, Jiangsu Province China; 4 Department of Central laboratory, Xuzhou Central Hospital, Jiangsu Xuzhou Jiefang South Road, No, Jiangsu, 199 China; 5 Department of Reproductive Medicine, Shanghai First Maternity and Infant Hospital, No. 2699 Gaoke West Road, Pudong District, Shanghai, China

**Keywords:** ERH gene, Bladder cancer, MYC gene, Migration and invasion

## Abstract

**Background:**

This study aimed to determine whether the enhancer of the rudimentary homolog (ERH) gene regulates cell migration and invasion in human bladder urothelial carcinoma (BUC) T24 cells and the underlying mechanism.

**Methods:**

First, we knocked down ERH in BUC T24 and 5637 cells by shRNA and then used wound healing cell scratch migration assays, transwell cell migration assays, transwell cell invasion chamber experiments and nude mouse tail vein transfer assays to determine the migration and invasion ability after ERH was knocked down. Moreover, we used gene expression profiling chip analysis and further functional experiments to explore the possible mechanism through which ERH knockdown downregulated metastasis ability in T24 cells.

**Results:**

Wound healing cell scratch migration assays, transwell cell migration assays, transwell cell invasion chamber experiments and nude mouse tail vein transfer assays all showed that the metastasis ability was significantly inhibited in human BUC T24 and 5637 cells with ERH knockdown. A gene expression profiling chip analysis in T24 cells showed that the MYC gene may be an important downstream target of the ERH gene, and the functional experiments showed that MYC is a functional target of ERH in BUC T24 cells.

**Conclusion:**

ERH knockdown could inhibit the metastasis of BUC T24 cells in vitro and in vivo. This study further explored the mechanism of the ERH gene in the metastasis of the T24 human bladder cancer cell line and found that ERH may regulate MYC gene expression. The results of this research provide a basis for the clinical application of ERH as a potential target for BUC treatment.

**Electronic supplementary material:**

The online version of this article (10.1186/s12885-019-5423-9) contains supplementary material, which is available to authorized users.

## Background

Bladder carcinoma is one of the most common urologic malignancies worldwide [1]. Bladder urothelial carcinoma (BUC) accounts for 90% of all bladder carcinomas, and 30% of BUCs are muscle-invasive bladder cancers [2]. BUC occurrence is second to only prostate cancer in the urothelial system: approximately 70,000 new cases occur each year and cause approximately 15,000 deaths [3]. Enhancer of rudimentary homolog (ERH), which has been found in plants, animals, and protists, may be involved in many cellular functions, such as pyrimidine metabolism, cell cycle progression, and transcription control [4]. In our previous study [5], we found ERH expression in human BUC 5637 and T24 cell lines and that this expression is upregulated in BUC cells. We showed that ERH knockdown inhibits cell proliferation and promotes apoptosis in BUC T24 cells. In this study, we further confirmed that the ERH gene regulates migration and invasion in human BUC 5637 and T24 cells. In addition, we used gene expression profile chips and found that ERH may regulate BC cell proliferation and apoptosis through the MYC gene.

## Methods

### Ethics statement, animals, cells and culture

All experiments were approved by the *Xuzhou Central Hospital Ethics Committee*. Animal experiments were performed following the protocols approved by the *Committee on Ethics of Animal Experiments* of the Animal Experiment Center of *Shanghai Sixth People’s Hospital*. Twenty nude mice purchased from the Animal Experiment Center of the *Shanghai Sixth People’s Hospital* were used in the experiments. The bladder cancer cell lines 5637 and T24 were purchased from the Cell Resource Center, *Shanghai Institutes for Biological Sciences* at *the Chinese Academy of Sciences*. The cells were cultured in RPMI 1640 medium containing 10% FBS, streptomycin, and penicillin at 37 °C in an incubator with 5% CO_2_.

### Reagents

Primary antibodies against Snail2 (# 9585), N-cadherin (# 13116), vimentin (# 3932) and E-cadherin (# 14472) were purchased from Cell Signaling Technology, and antibodies against fibronectin (ab6328) and Twist (ab49254) were purchased from Abcam. Fetal bovine serum (FBS, A11–102) was purchased from the Ausbian Corporation. Dulbecco’s minimum essential medium (DMEM, 10–013-CVR) and transwell migration (3422) and invasion (354480) kits were all purchased from Corning. D-Luciferin (40902ES01) was purchased from Shanghai Qianchen Biotechnology Co., Ltd. Trypsin (T0458-50G) and PBS (1710584) were purchased from Bioengineering (Shanghai) Co., Ltd. D-Hanks was prepared by Shanghai Genechem Technology Co., Ltd. Sigma provided the Giemsa staining solution (32884) and sodium pentobarbital (P3761). Shanghai First Chemical Reagent Co., Ltd. supplied the dimethyl sulfoxide (DMSO, 130701). GAPDH (sc-32,233) and the secondary goat anti-mouse IgG antibody (sc-2005) were purchased from Santa Cruz Biotechnology. A BCA protein assay kit (P0010S) and RIPA lysate (strong) (P0013B) were purchased from Pik Wan Company. RIPA lysate (WB-0071) and NP-40 lysate (P0013F) were purchased from Shanghai Dingguo Biotechnology Co. Prestained protein markers (00161543) and an ECL-PLUS/Kit (M3121/1859022) were provided by Thermo. Carestream Health supplied the medical X-ray film (038401501). X-ray film developer powder and fixing powder (P61–04-1) were purchased from the Shanghai Guanlong Photographic Material Factory.

### Production of ERH knockdown (KD) cells

BUC cells with ERH knockdown were prepared as described in a previous article [5]. A lentiviral-based shRNA strategy was used to knockdown ERH in human bladder cancer T24 cells. Lentiviruses expressing shRNA specifically targeting human ERH (ERH-shRNA, ERH KD group, target sequence: CTGGTTTACCGAGCTGATA) or a scrambled sequence (ScrshRNA, ERH normal cell (NC) group, sequence: TTCTCCGAACGTGTCACGT) were generated. Subsequently, T24 human bladder cancer cells were used to examine the knockdown efficiency at the mRNA and protein levels. The cells were plated in 6-well plates and subsequently infected with the lentivirus expressing ERH-shRNA. The cells were cultured at 37 °C in an incubator with 5% CO_2_ until they reached 30% confluence. The cells were harvested, and total RNA was extracted to determine the knockdown efficiency using real-time PCR assays. When the infection efficiency was greater than 70%, the cells were used for the subsequent experiments. The results showed that the lentiviral-based shRNA strategy could effectively and efficiently inhibit ERH expression at the mRNA level (Additional file 1).

### Wound healing cell scratch migration assay

Cell migration was assessed by wound healing cell scratch migration assays. BUC 5637 and T24 cells were cultured for 24 h and then diluted to 4000 cells/ml by microscopic cell counting. After the cells were well mixed, they were added to 6-well plates and divided into ERH NC and ERH KD groups. When the cells were 80–90% confluent, the cell monolayer was scraped with a sterile 200-μl pipette tip. The cells were washed gently with PBS 3 times. After incubation for 0 h, 3 h, 6 h, and 24 h, the cells were fixed, and images were taken. The migration distance and mobility were calculated using Python-based image analysis software named Python(x, y) (v1.0, Shanghai Genechem Co., Ltd.).

### Transwell cell migration assay

Transwell chambers were used to investigate cell migration. Cells were divided into ERH NC and ERH KD groups, and the BUC 5637 and T24 cells were starved for 24 h. After digestion and centrifugation, the cells were resuspended in serum-free medium, and the cells were counted under a microscope. The cell concentration was adjusted to 1 × 10^5^ cells/ml, and a 100 μl/well cell suspension was placed in the bottom of a 24-well plate. We added 600 μl of 30% FBS medium to each well and then added the cell suspension to an MTS 96-well plate. We added 5000 cells to each well and measured the absorbance at OD570 as a metastasis reference. After incubation at 37 °C for 24 h, the cells in the internal compartment were removed with a cotton swab. The migrated cells were fixed in methanol and stained with a 1% crystal violet solution. Under a light microscope, three randomly selected 200× fields of view were counted, and images were captured.

### Transwell cell invasion chamber experiments

Transwell invasive chambers were used to determine cell invasion. Cells were divided into ERH NC and ERH KD groups. BUC 5637 and T24 cells were plated in new 24-well plates, 500 μl of serum-free medium was added to the upper and lower chambers, and a serum-free cell suspension was prepared after culture at 37 °C for 2 h. The cells were counted under a microscope and were then adjusted to 1 × 10^5^ cells/ml. The cells were all transferred to a new well plate, the upper chamber medium was removed, 500 μl of the cell suspension was added, and the lower chamber was filled with 750 μl of 30% FBS medium. At the same time, a 96-well MTS plate was plated with the cell suspension; 5000 cells/well were used, and the absorbance at OD570 was determined as a reference for metastasis after inoculation. After culture at 37 °C for 22 h, the cells in the inner chamber were not wiped off with a cotton swab. The invasive cells were fixed and stained with Giemsa stain. Under a light microscope, three fields (200×) were selected randomly for counting and photographing to compare the differences in cell invasiveness between the two groups.

### Western blotting

The cells were divided into 4 groups: 5637 ERH NC, 5637 ERH KD, T24 ERH NC and T24 ERH KD groups. After culture for 48 h, the cells were washed twice with precooled PBS and lysed with 200 μl of RIPA lysis buffer. The cells were lysed for 15 min, sonicated and centrifuged to collect the supernatants. Total protein was quantified by the BCA method. We used 30–50 μg of the total protein samples for SDS-PAGE (separation gel concentration 10%). The proteins were transferred onto PVDF membranes, and Western blotting was then performed. The cells were blocked with blocking solution (TBST solution containing 5% nonfat milk) for 1 h at room temperature. E-Cadherin, fibronectin, Twist, vimentin, Snail2, and GAPDH primary antibodies diluted in blocking solution were added separately. The membranes were washed 4 times with TBST for 8 min each. Fluorescein-labeled goat anti-mouse IgG or goat anti-rabbit IgG diluted in blocking solution was added. The PVDF membranes were incubated at room temperature for 1.5 h, blocked with TBST, and then washed 4 times for 8 min. We used the ECL method combined with X-ray imaging to visualize the proteins.

### Nude mouse tail vein transfer assay

We performed a nude mouse tail vein transfer assay to observe tumor metastasis in mice in vivo by fluorescence imaging. We divided BUC T24 cells into ERH NC-luciferase and ERH KD-luciferase groups. Tumor cell suspensions were prepared at a concentration of 2 × 10^7^ cells/mL, and nude mice were injected with a 200-μl cell suspension through the tail vein. A bioluminescent image (BLI) scan was performed once per week. Before BLI observation, the mice were anesthetized with an intraperitoneal injection of 0.7% pentobarbital sodium at 10 μl/g. Fluorescence was observed and quantitatively analyzed. The mice were euthanized, and their lungs were harvested after 28 days for imaging.

### Gene expression profiling chip analysis

We further analyzed and compared gene expression profiling chip data for the ERH NC and ERH KD groups and found that the gene profiles were significantly different. Screening criteria were required to meet |FC| > 2 and *P*-value < 0.05. Molecule network analysis was performed for significant differential genes based on Ingenuity® Pathway Analysis (IPA®) [6]. IPA is an analysis and search tool that determines the significance of ‘omics data’ and identifies new targets or candidate biomarkers within the context of biological systems.

### Statistical analysis

Statistical analyses were performed using SAS 9.43 (SAS Institute Inc., Cary, NC, USA). Significant differences in continuous data (mean ± SD) were estimated using Student’s *t* test. The data were analyzed by one-way ANOVA. For all analyses, *p* < 0.05 (*) and *p* < 0.01 (**) were defined as statistically significant differences.

## Results

### Wound healing cell scratch migration assay

**Fig. 1:**
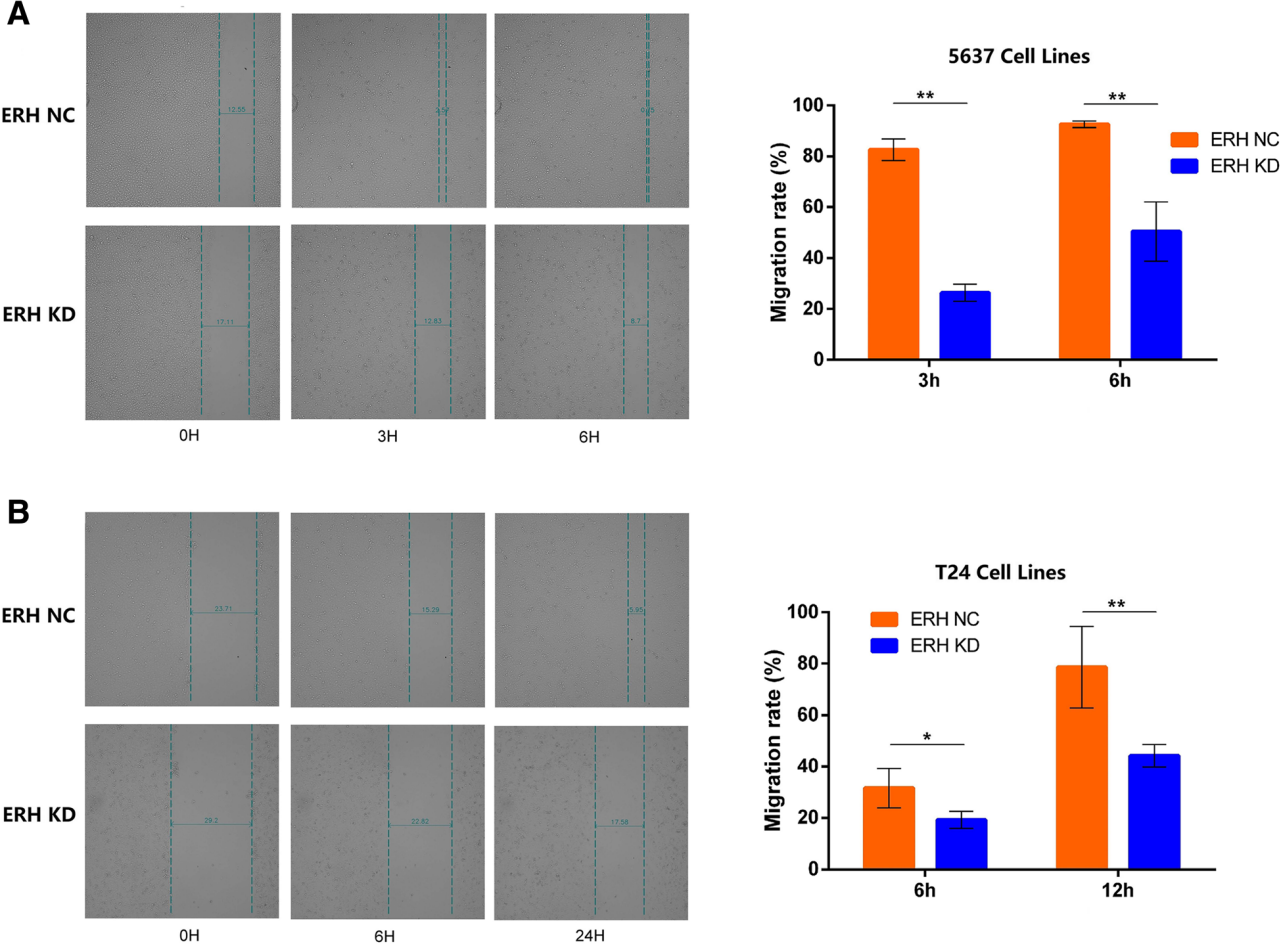
Wound healing cell scratch migration assay data. **a**. There were significant differences in the metastasis rates of BUC 5637 cells at 3 h and 6 h between the ERH NC and ERH KD groups. (**p < 0.01); **b**, BUC T24 cells at 6 h in the ERH KD and ERH NC groups (*p < 0.05). The differences were statistically significant at 24 h between the ERH NC and ERH KD groups (**p < 0.01)

Wound healing cell scratch migration assays were used to assess whether ERH knockdown inhibited the migration of BUC 5637 and T24 cells (Table 1). As shown in Fig. 1, cells migrated to the wound site 24 h after ERH knockdown, and the cell migration rate was significantly reduced.

**Table 1:** Wound healing cell scratch migration assay

		Group	Migration rate(%)	Variance	t Value	*p* Value
5637 Cells	3 h	ERH NC	82.6 ± 4.3	Equal	22.91	< 0.0001
		ERH KD	26.4 ± 3.4			
	6 h	ERH NC	92.6 ± 1.3	Unequal	8.09	0.0011
		ERH KD	50.4 ± 11.6			
T24 Cells	6 h	ERH NC	31.6 ± 7.7	Equal	3.33	0.0104
		ERH KD	19.2 ± 3.3			
	24 h	ERH NC	78.6 ± 15.9	Unequal	4.68	0.0067
		ERH KD	44.2 ± 4.4			

There were significant differences in the metastasis rates of BUC 5637 cells at 3 h and 6 h between ERH-knockdown (ERH KD) and control group (ERH NC) (p < 0.01); There were significant differences in the metastasis rates of BUC T24 cells at 6 h between ERH KD and ERH NC group (p < 0.05) and 24 h (p < 0.01)

### Human bladder 5637 and T24 cell migration was downregulated by ERH knockdown

**Fig. 2:**
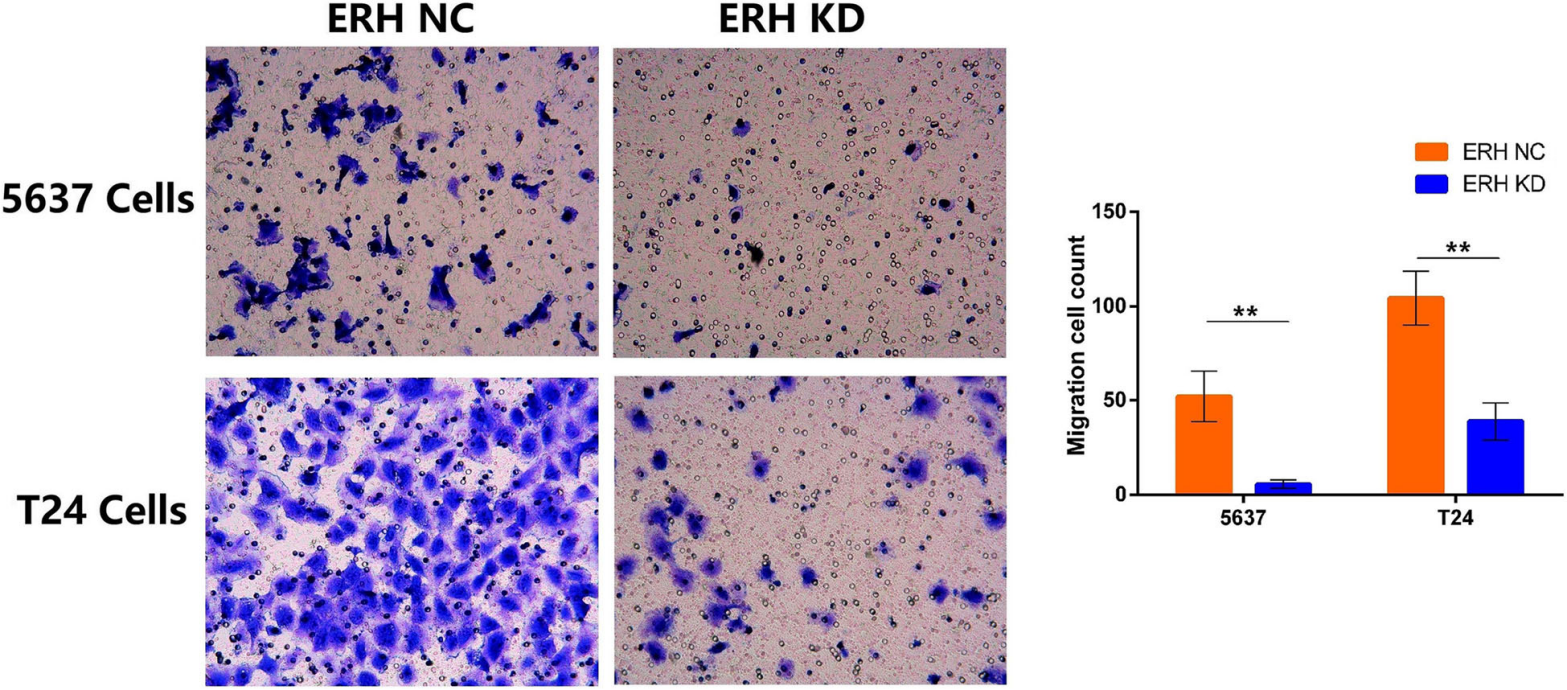
Transwell migration chamber assay. Transwell migration assay results for BUC 5637 and T24 cells in the ERH NC and ERH KD groups (200×). The visible cell number of the ERH KD group was significantly less than that of the ERH NC group (**p < 0.01)

As shown in Fig. 2, the migration cell counts for BUC 5637 and T24 cells were all statistically significant between the ERH NC and ERH KD groups (*p* < 0.05). A transwell migration chamber assay was used, and we found that knockdown of the ERH gene can inhibit the migration of BUC 5637 and T24 cells (Table 2, *p* < 0.01).

**Table 2:** Transwell migration chamber assay

BUC	Group	Migration cell count	Variance	t Value	*p* Value
5637 cells	ERH NC	52.15 ± 13.47	Unequal	17.64	< 0.0001
	ERH KD	5.74 ± 2.36			
T24 cells	ERH NC	104.26 ± 12.24	Unequal	21.68	< 0.0001
	ERH KD	38.96 ± 9.75			

The differences in cell counts of BUC 5637 and T24 were all statistically significant between ERH KD and NC groups (p < 0.01)

### Human bladder 5637 and T24 cell invasion was downregulated by ERH knockdown

**Fig. 3:**
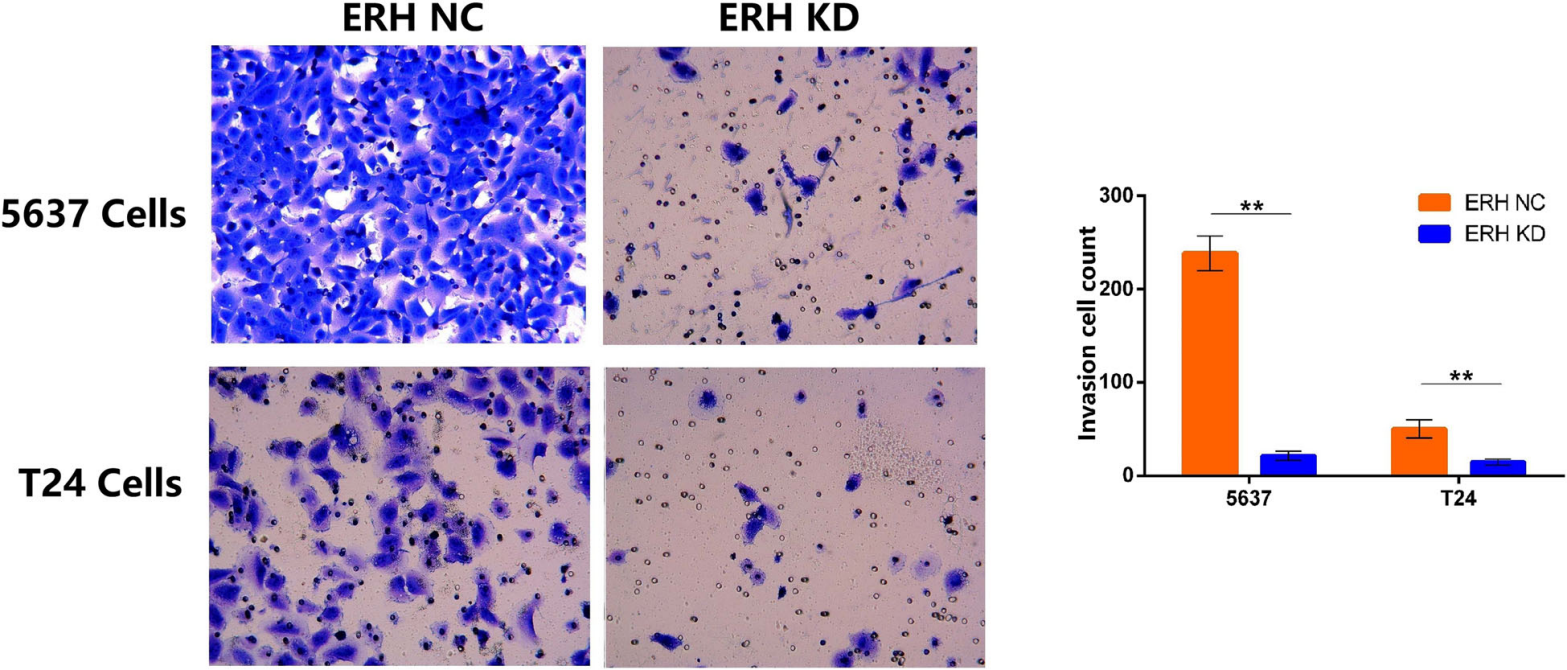
Transwell invasion chamber assay. Transwell invasion chamber assay results for BUC 5637 and T24 cells in the ERH NC and ERH KD groups (200×). The visible cell number of the ERH KD group was significantly less than that of the ERH NC group (**p < 0.01)

As shown in Fig. 3, the invasion cell counts of BUC 5637 and T24 cells were all significantly different (*p* < 0.05). A transwell invasion chamber assay was used, and we found that knockdown of the ERH gene can inhibit the invasion of BUC 5637 and T24 cells (Table 3, *p* < 0.01).

**Table 3:** Transwell invasion chamber assay

BUC	Group	Invasion cell count	Variance	t Value	*p* Value
5637 cells	ERH NC	238.52 ± 18.29	Unequal	59.56	< 0.0001
	ERH KD	21.30 ± 4.97			
T24 cells	ERH NC	50.07 ± 9.65	Unequal	18.12	< 0.0001
	ERH KD	14.56 ± 3.26			

The differences in cell counts of BUC 5637 and T24 were all statistically significant between ERH KD and ERH NC groups (p < 0.01)

### The regulation of EMT (epithelial–mesenchymal transition) marker proteins by ERH

**Fig. 4:**
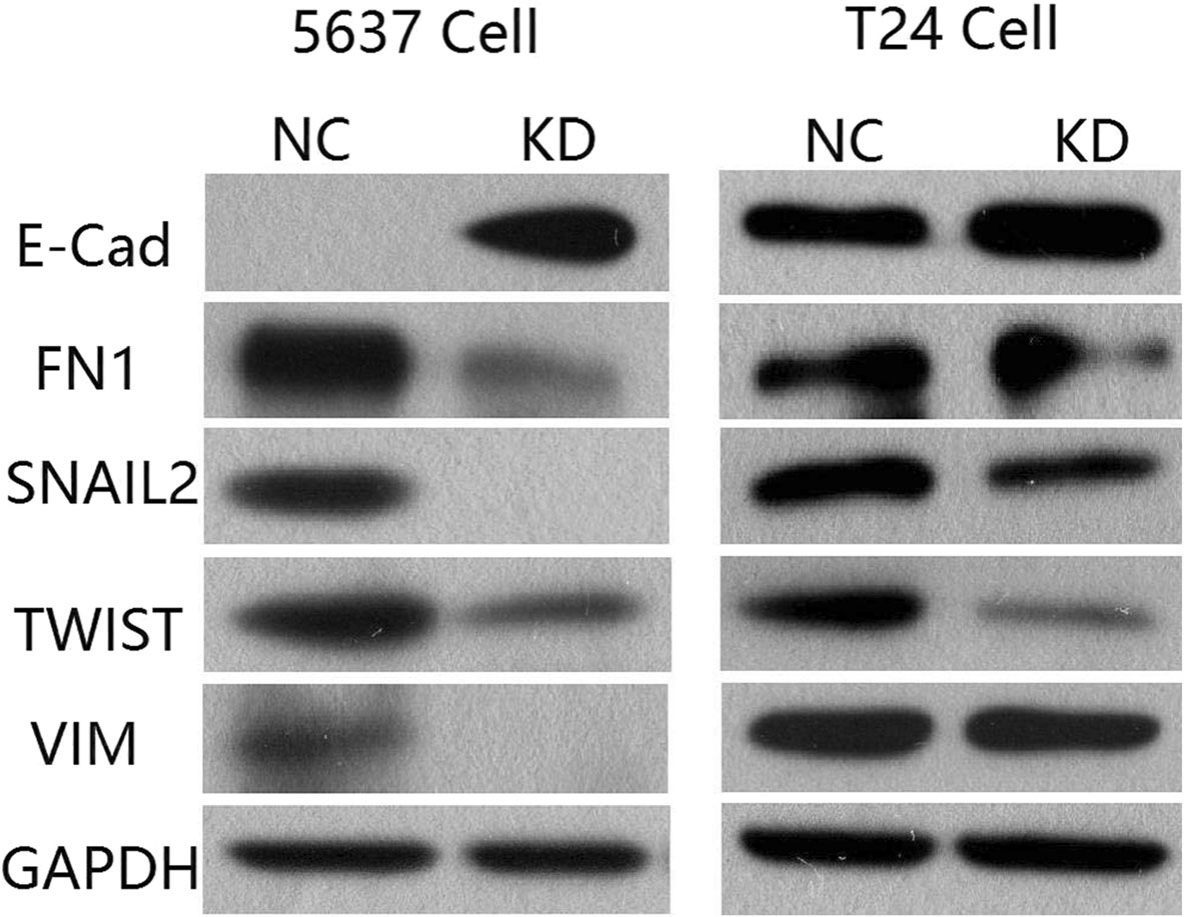
Detection of EMT marker proteins in BUC 5637 and T24 cells by Western blotting. Western blotting was used to detect the EMT marker proteins in BUC 5637 and T24 cells. E-Cadherin expression was significantly increased in BUC 5637 and T24 cells after ERH knockdown, while the expression of fibronectin, Twist, vimentin and Snail2 was significantly decreased

Western blotting was used to detect the migration-related proteins of BUC 5637 and T24 cells. As shown in Fig. 4, E-cadherin expression was significantly increased in BUC 5637 and T24 cells after ERH knockdown, while the expression of fibronectin, Twist, vimentin and Snail2 was significantly decreased.

### Nude mouse tail vein transfer assays showed that ERH knockdown could inhibit the lung metastasis of BUC T24 cells

**Fig. 5:**
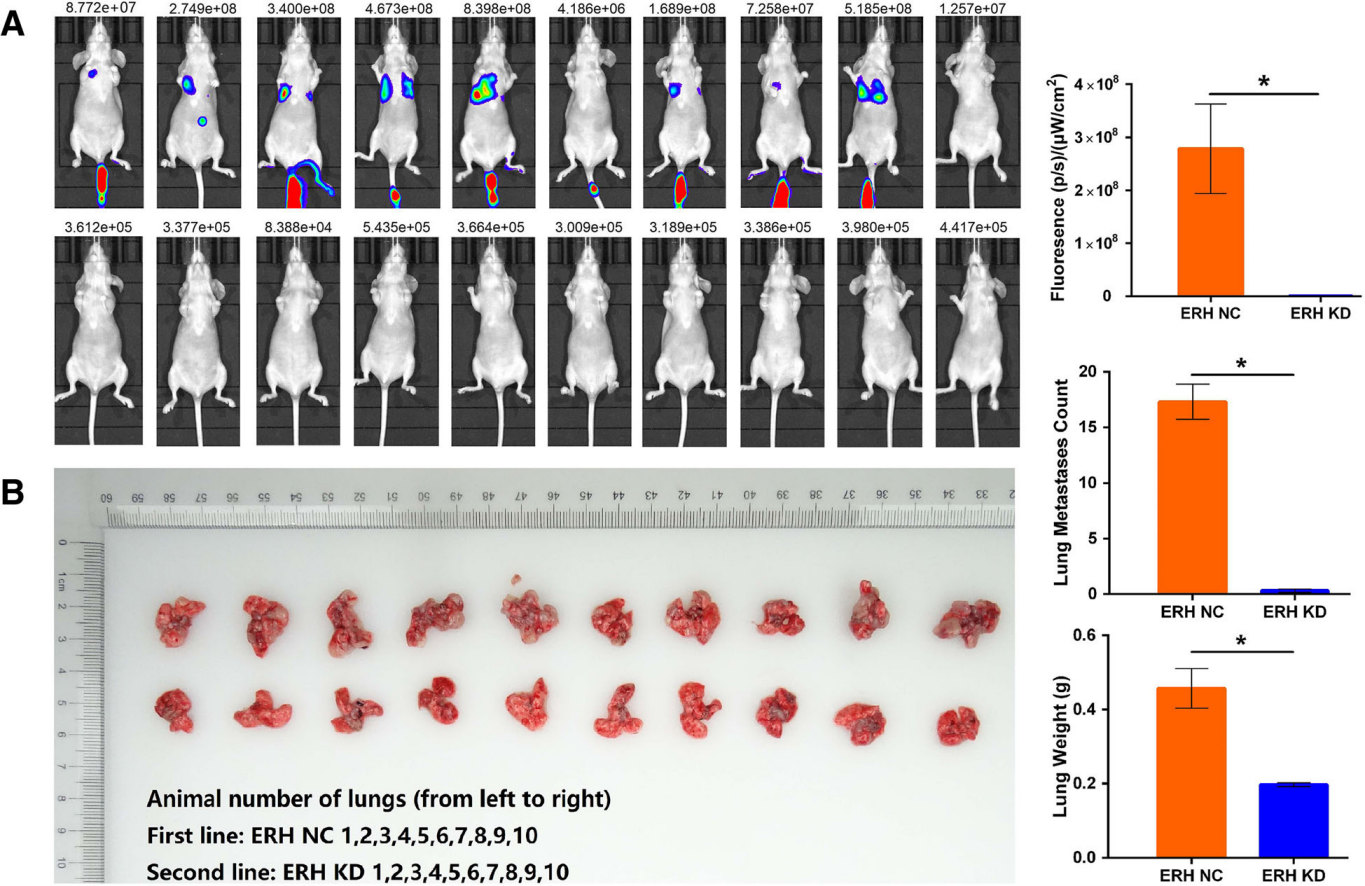
**a** Twenty-eight days after the tail vein injection of ERH NC-luciferase or ERH KD-luciferase BUC T24 cells, whole body BLI was performed. **b** The lungs from mice injected with either ERH NC-luciferase or ERH KD-luciferase BUC T24 cells were imaged after euthanization

We injected ERH NC-luciferase and ERH KD-luciferase BUC T24 cells via the tail vein to examine whether ERH knockdown could affect BUC metastasis in vivo. Twenty-eight days after transplantation, the mice were euthanized, and their lungs were harvested. By ex vivo imaging, we further confirmed that ERH knockdown could suppress lung metastasis and vessel invasion in BUC T24 cell lines (Fig. 5a). The number of lung metastases was judged by the amount of fluorescence and by manually counting the lung nodule number. Lung metastases were detected in 10/10 mice in the ERH NC-luciferase group and in 3/10 mice in the ERH KD-luciferase group (Fig. 5b). The number of lung metastases in the ERH NC-luciferase group was 17.3 ± 1.59, and the number of lung metastases in the ERH KD-luciferase group was 0.30 ± 0.15 (T = 10.68, *p* < 0.01). The lung weight of the ERH NC-luciferase group was 0.46 ± 0.17 g, and that of the ERH KD-luciferase group was 0.20 ± 0.02 g (T = 4.85, *p* < 0.01).

### Silencing ERH alters oncogene expression in BUC cells

**Fig. 6:**
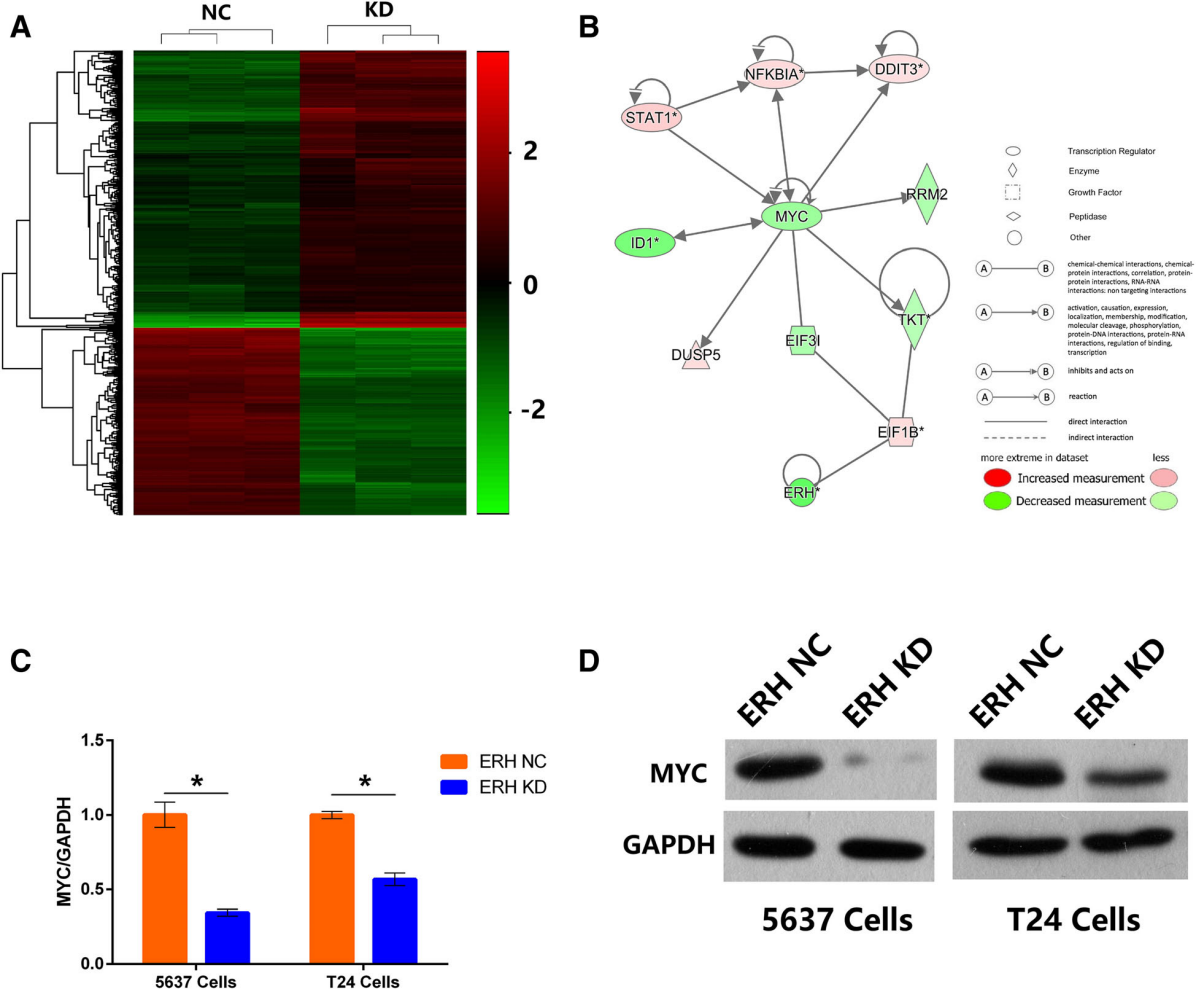
**a** Hierarchical cluster analysis of ERH KD and NC cells. Heatmap colors represent the mean-centered fold change expression in log-space. **b**. According to the results of the ERH microarray, several differentially expressed genes related to cell migration and invasion were selected. A network map of these genes shows that the MYC gene was in the center position. **c** shows the expression of the MYC gene after ERH knockdown in BUC 5637 and T24 cells using the real-time PCR method. MYC gene expression in the ERH KD group was 0.345 and 0.568 times that of the ERH NC group in 5637 and T24 cells, respectively (all *p* < 0.01). **d** shows the Western blot analysis of MYC protein expression in BUC 5637 and T24 cells following ERH knockdown. The expression of MYC protein in the ERH KD group was significantly lower than that in the ERH NC group

To further explore the molecular mechanism of ERH in affecting the migration and invasion of BUC cells, whole-genome Affymetrix GeneChip hybridization was adopted to discover changes in gene expression induced by ERH knockdown and the potential regulatory mechanisms. Following bioinformatic and normalization analyses, the two groups (ERH NC and ERH KD) showed differences according to a hierarchical cluster analysis, as shown in Fig. 6. Gene expression microarray results showed that the expression of the ERH gene in the ERH KD group was significantly reduced; the FC value was − 4.50, *p* < 0.0001. Compared to those in the ERH NC group, 344 genes were upregulated, and 254 genes were downregulated in the ERH KD group (Additional file 2, Fig. 6a).

To further investigate the related regulation network of ERH-induced malignancy, we analyzed the knowledge-based interactome and constructed a network surrounding ERH using IPA. According to the network we constructed, MYC seems to be an important downstream target of ERH. Based on this result, we speculate that the MYC gene may be an important downstream target of the ERH gene, so we further confirmed the functional relationship and regulation of the MYC and ERH genes through functional recovery experiments (Fig. 6b).

### MYC was downregulated by ERH knockdown

The expression of the MYC gene in the BUC 5637 and T24 cell lines was detected by real-time PCR and Western blotting after ERH gene knockdown.

Real-time PCR was used to detect the expression of the MYC gene in BUC 5637 and T24 cells after ERH knockdown. MYC gene expression in the ERH KD group was 0.345 and 0.568 times that of the ERH NC group in 5637 and T24 cells, respectively. These differences were statistically significant and indicated that MYC gene expression was significantly decreased in the ERH KD group (Fig. 6c, all *p* < 0.01).

The expression of the MYC protein in BUC 5637 and T24 cells after ERH knockdown was detected by Western blotting. From the results (Fig. 6d), it can be seen that MYC protein expression was significantly lower in the ERH KD group than in the NC group in 5637 and T24 cells.

### MYC is a functional target of ERH in the BUC T24 cell line

The 5637 and T24 BUC cell lines were used to determine whether the MYC downregulation induced by ERH knockdown contributed to the inhibition of migration and invasion. We inhibited ERH expression in human bladder 5637 and T24 cells with or without MYC overexpression and used normal ERH cells as a control. The cells were divided into four groups: ERH NC, MYC NC, ERH KD, and MYC OE (overexpression). The invasion and metastasis of the four groups for the 5637 and T24 cell lines were determined by transwell experiments and wound healing cell scratch migration assays.

**Fig. 7:**
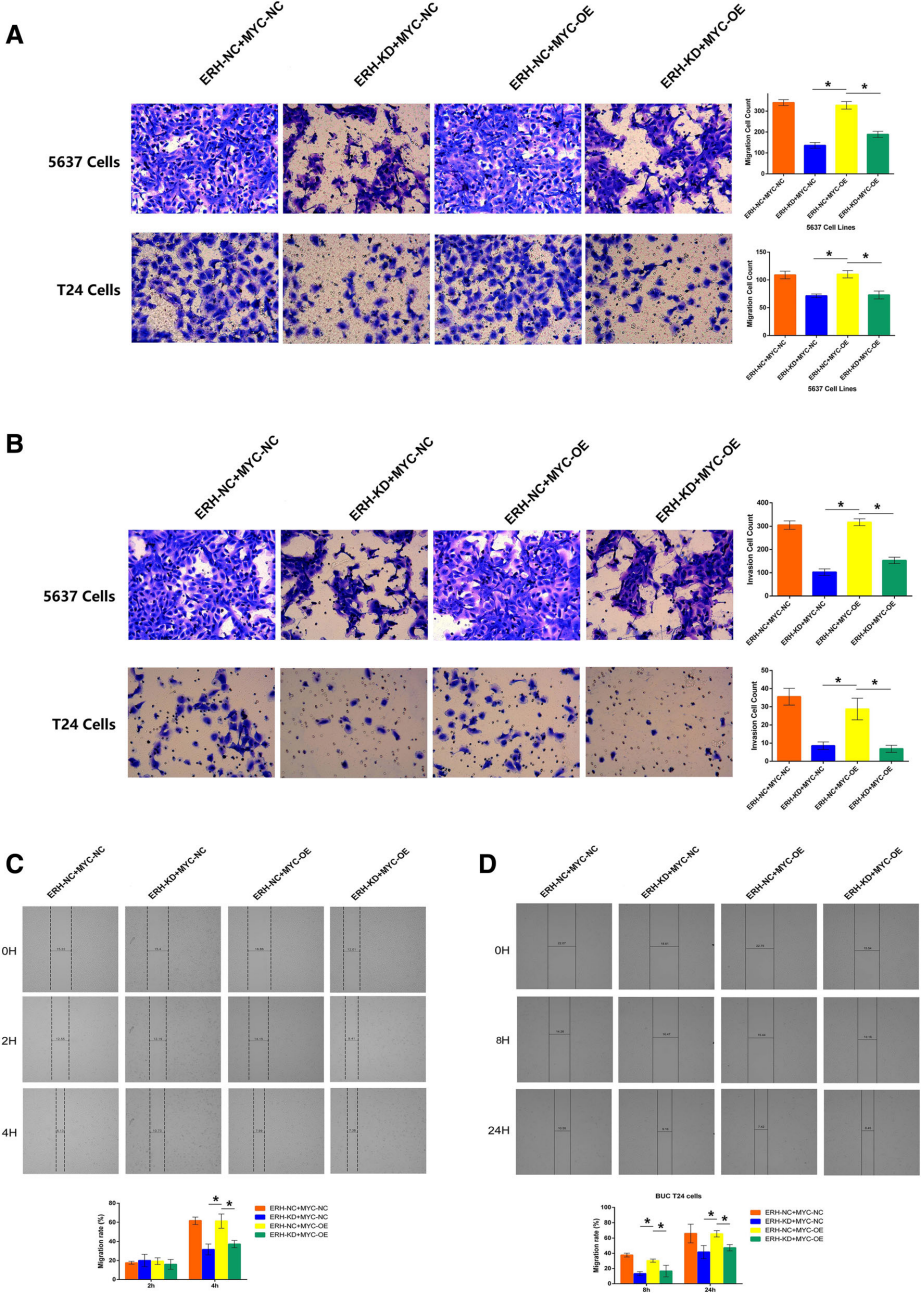
Shows the functional rescue experiment results after ERH was knocked down and MYC was overexpressed or not. **a** shows the transwell migration assays in BUC 5637 and T24 cells (200X); the results showed that the overexpression of MYC inhibited the dampening effect of ERH knockdown on BUC 5637 and T24 cell migration. **b** shows the transwell invasion assays (200X), and these results were the same as the transwell migration assay results. **c** and **d** shows the wound healing assay results for BUC 5637 and T24 cells after ERH knockdown or MYC overexpression. The results show that the overexpression of MYC partially inhibited the dampening effect of ERH knockdown on BUC 5637 and T24 cell migration and invasion

As shown in Fig. 7, the downregulation of the ERH gene inhibited the migration and invasion of BUC 5637 and T24 cells. The overexpression of MYC partially inhibited the dampening effect of ERH knockdown on BUC 5637 and T24 cell migration and invasion.

## Discussion

The ERH gene, which was first found in Drosophila, can also be found in flowering plants, *Aedes aegypti*, house mice, nematodes and humans, and its function has been implicated in pyrimidine biosynthesis and the cell cycle [4]. The ERH monomer comprises a single domain consisting of an antiparallel β-sheet (β1, β2, β3, and β4) and three α-helices (α1, α2, and α3) [7]. Many of the surface residues of ERH are conserved, including patches of hydrophobic and charged residues, suggesting protein-protein interaction interfaces [8]. The structure of ERH is characterized by a novel α + β fold, which is a four-strand antiparallel β-sheet with three alpha-helixes on one side of the sheet. The β-sheets from the two monomers together constitute a pseudo-β-barrel and form the center of the functional ERH dimer, with a cavity channel at the dimer interface; ERH has also been shown to be important in pyrimidine biosynthesis, cell cycle regulation, and transcriptional repression [9]. ERH was shown to be an interaction partner of DCoH/PCD (dimerization cofactor of HNF1/pterin-4-alpha-carbinolamine dehydratase) by a yeast two-hybrid assay. DCoH/PCD is a multifunctional factor originally identified as a positive cofactor of the HNF1 homeobox transcription factors [10].

In 2008, it was shown that the expression of ERH is clearly higher in malignant breast cells than in benign breast cells in both primary human breast cancer and cell models of breast cancer [11]. ERH is known to interact with the zinc-finger protein CIZ1, a promoter of DNA replication that interacts with p21 (Cip1), a CDK2 inhibitor critical for cell cycle regulation [12]. Moreover, ERH interacts with SKAR, a cell growth regulator in the S6K1-signaling pathway [13]. ERH also interacts with the H19 gene and regulates growth in nephroblasts, rhabdomyosarcomas and choriocarcinoma cells [14]. ERH interacts with the spliceosome protein SNRPD3 and is required for the mRNA splicing of the mitotic motor protein CENP-E [15]. In addition, ERH expression is inversely associated with the survival of colorectal cancer patients whose tumors harbor KRAS mutations [16]. A predicted target site was detected in the 3′-untranslated region of the ERH gene, and transfected cells showed an interaction between the luciferase reporter containing the target sequences and miR-574-3p in lung cancer A549 cell lines [17]. In 2015, it was shown that ERH regulates the DNA damage response in hepatocellular carcinoma [18]. In all of these previous studies, the relationship between the ERH gene and tumor migration and invasion was not studied.

In our previous study [5], we found that ERH is positively expressed in human BUC 5637 and T24 cell lines and is upregulated in BUC cells. We further studied the relationship between ERH and tumor cell migration and invasion. After ERH was knocked down, human BUC 5637 and T24 cell migration and invasion decreased significantly. There is no existing report about the correlation between the ERH gene and the migration and invasion of BUC cells. We used a wound healing assay to determine whether ERH knockdown inhibited the migration of BUC 5637 and T24 cells. The results showed that the cell migration rate was significantly decreased after ERH knockdown. Transwell chamber assays were used to evaluate whether ERH knockdown could inhibit the migration of BUC 5637 and T24 cells. The results indicated that there were significant differences in the numbers of migrated BUC 5637 and T24 cells. Transwell invasion cell assays were used to evaluate whether ERH knockdown could inhibit the invasiveness of BUC 5637 and T24 cells. A nude mouse tail vein transfer assay showed that ERH knockdown could inhibit the metastasis of BUC T24 cells. These results show that the differences in the invasiveness of BUC 5637 and T24 cells after ERH knockdown are significantly different.

EMT is crucial in cancer cell migration and invasion [19]. Three major changes constitute EMT in cancer cell migration. First, epithelial cells lose both cell-cell adhesions and the expression of the epithelial cell marker E-cadherin. Second, these cells acquire the expression of N-cadherin, a mesenchymal marker. Third, the cells undergo major cytoskeletal rearrangements that enable them to acquire mesenchymal properties, such as cell migratory behaviors [20]. EMT is a potent component of the signaling and cellular alterations involved in the invasive growth and metastasis of cancer cells [21]. Zhao et al. [22] found that Twist and vimentin correlated with grade, recurrence, and progression but not with stage, and E-cadherin was related to stage but not the other parameters. Their study showed that vimentin is a potential independent indicator for predicting BUC progression and survival. Hong et al. [23] revealed that Snail/Twist signaling might be required for TGF-β-induced EMT in bladder cancer cells. Western blotting was used to detect the migration-related proteins in BUC 5637 and T24 cells. The expression of E-cadherin in BUC 5637 cells and T24 cells after ERH knockdown was significantly increased, while the expression of fibronectin, Twist, vimentin and Snail2 was significantly decreased. This suggests that ERH may affect the migration and invasion ability of BUC through the EMT process. The present study indicated that the expression of E-cadherin was related to ERH knockdown in 5637 and T24 cells; these results agree with other studies showing that the loss of E-cadherin expression has been associated with migration and invasion in numerous types of epithelium-derived cancer cells [24]. Gene expression changes that help inhibit the epithelial phenotype and activate the mesenchymal phenotype involve the regulatory factors Snail and Twist [25]. The results of the present study also showed that the expression of Snail and Twist was related to ERH knockdown, which indicated that ERH participates in the EMT process. Finding the downstream target gene of ERH is key to studying the mechanism through which ERH affects cell migration and invasion through the EMT process.

Thus, we explored the molecular biological mechanism through which the ERH gene affects the migration and invasion of BUC cells by a gene expression profile chip. The gene expression profile chip showed that the expression of the ERH gene in the ERH knockdown group was significantly reduced, and the FC value was − 4.50, *p* < 0.0001. Compared with those in the NC group, 344 genes were upregulated, and 254 genes were downregulated in the ERH knockdown group. We next built a network map based on IPA and found that the MYC gene might be an important downstream target of ERH.

The MYC gene belongs to a group of early discovered classical oncogenes, including c-MYC, n-MYC and l-MYC, which are located on chromosome 8, chromosome 2 and chromosome 1, respectively. Over the past 30 years, MYC has attracted the attention of a large number of cancer scientists, virus experts, biochemists and geneticists for its role in promoting proliferation, progression, chemoresistance, angiogenesis and tumor metastasis [26]. MYC promotes metastasis in established animal models of lung and prostate cancer [27, 28]. It has been proven that c-MYC transforms human mammary epithelial cells through the repression of the Wnt inhibitors DKK1 and SFRP1 in breast cancer cell lines [29]. E-cadherin repression contributes to c-Myc-induced epithelial cell transformation [30], which is consistent with our experimental results.

In this study, we inhibited ERH expression in human bladder T24 cells with or without MYC overexpression to determine whether ERH-mediated MYC downregulation contributed to the inhibition of migration and invasion. We found that ERH knockdown does inhibit migration and invasion through MYC in BUC T24 cells through functional recovery experiments. Therefore, targeting the ERH-MYC-EMT regulatory circuit may be a novel strategy for the treatment of bladder cancer.

The migration and invasion of BUC is the most critical factor affecting prognosis. Thus, it is urgent to find the underlying mechanism to identify a prognostic biomarker that can supply a potential therapeutic target. This study identified and explored the role of the ERH gene in the migration and invasion of BUC and the possible mechanism. This may provide a basis for the clinical application of ERH as a target for the detection and treatment of bladder cancer, which has great clinical potential.

## Conclusions

In this article, we report for the first time that ERH knockdown inhibits the migration and invasion of BUC 5637 and T24 cells in vitro and in vivo. This study explores the expression and mechanism of the ERH gene in metastasis in the human bladder 5637 and T24 cell lines. We found that ERH knockdown does inhibit migration and invasion through MYC in BUC T24 cells. This study provides novel insight into the clinical application of ERH as a target for BUC treatment, which has great potential for clinical application.

## Additional file

Additional file 1: ERH expression after ERH knockdown. Shows the ERH expression after ERH knockdown evaluated by qRCR. The results show that there were significant differences of ERH/GAPDH between the ERH NC and ERH KD groups for both 5637 and T24 cells (*p < 0.05). (JPG 227 kb)

Additional file 2: Different 598 genes after ERH knockdown in bladder cancer T24 cells. Shows all of the 598 genes for which an absolute fold changes higher than 2 after ERH was knockdown in bladder cancer T24 cells. (XLSX 107 kb)

## Abbreviations

BLI: Bioluminescent image
BUC: Bladder urothelial carcinoma
DCoH/PCD: Dimerization cofactor of HNF1/pterin-4-alpha-carbinolamine dehydratase
DMSO: Dimethyl sulfoxide
EMT: Epithelial–mesenchymal transition
ERH gene: The enhancer of the rudimentary homolog gene
IPA®: Ingenuity® Pathway Analysis
KD: Knockdown
NC: Normal cell
