# Supplementary figures and images for: The ERH gene regulates migration and invasion in 5637 and T24 bladder cancer cells
Source: BMC Cancer. 2019 Mar 12;19:225. doi: 10.1186/s12885-019-5423-9 (PMC6417071; doi:10.1186/s12885-019-5423-9)

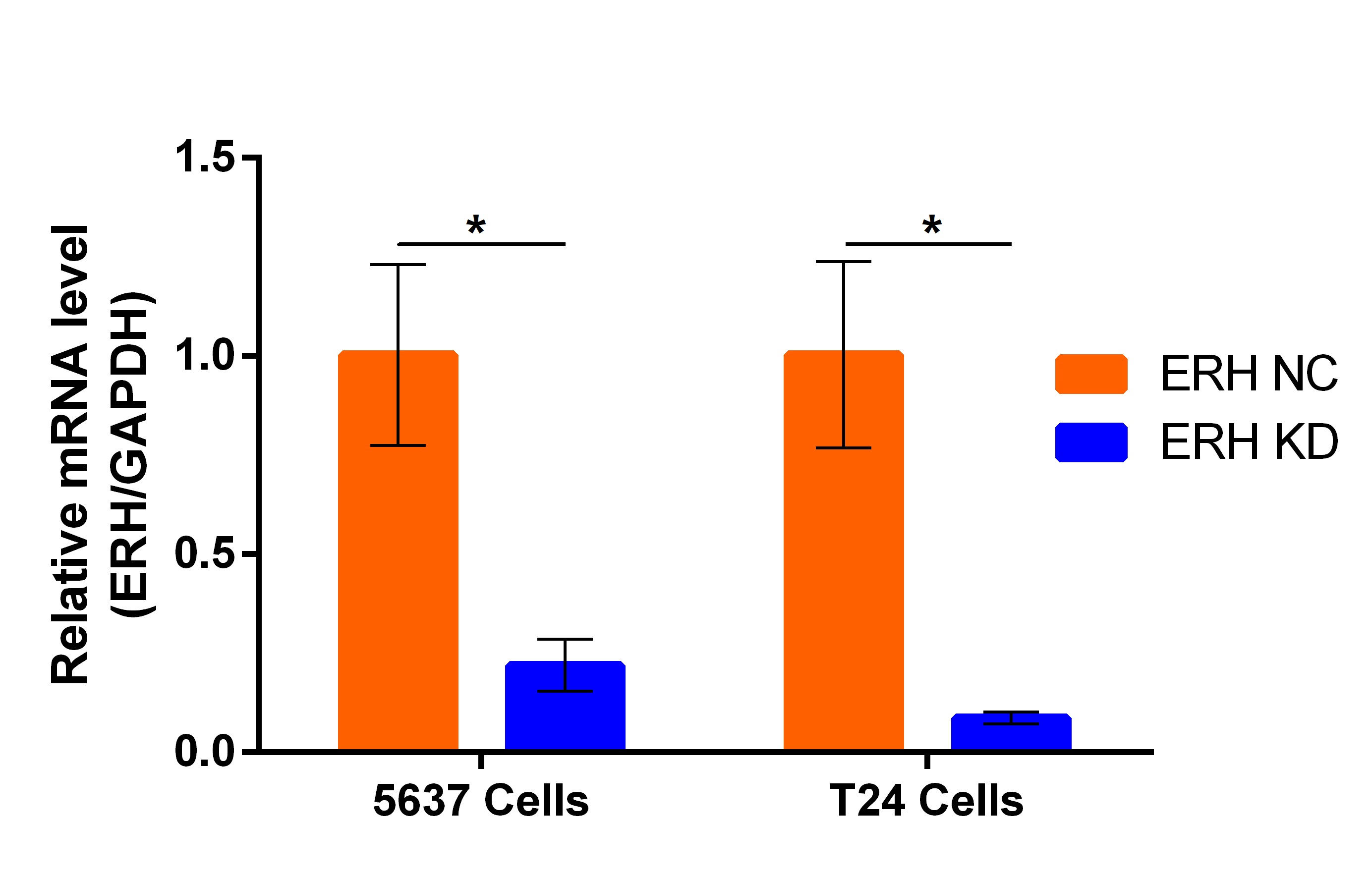

Supplement: Supplementary file 1 — ERH expression after ERH knockdown. Shows the ERH expression after ERH knockdown evaluated by qRCR. The results show that there were significant differences of ERH/GAPDH between the ERH NC and ERH KD groups for both 5637 and T24 cells (*p < 0.05). (JPG 227 kb) [file 12885_2019_5423_MOESM1_ESM.jpg]
